# Clozapine Can Be the Good Option in Resistant Mania

**DOI:** 10.1155/2016/3081704

**Published:** 2016-07-25

**Authors:** S. M. Yasir Arafat, S. M. Atikur Rahman, Md. Maruful Haque, Mohsin Ali Shah, Sultana Algin, Jhunu Shamsun Nahar

**Affiliations:** Department of Psychiatry, Bangabandhu Sheikh Mujib Medical University, Shahbag, Dhaka 1000, Bangladesh

## Abstract

Bipolar mood disorder is a mental disorder with a lifetime prevalence rate of about 1% in the general population and there are still a proportion of individuals who suffer from bipolar mood disorders that are resistant to standard treatment. Reporting clozapine responsive mania that was not responding to two previous consecutive atypical antipsychotics and one typical antipsychotic was aimed at. A 17-year-old male manic patient was admitted into the psychiatry inpatient department and was nonresponsive to Risperidone 12 mg daily for 4 weeks, Olanzapine 30 mg daily for 3 weeks, and Haloperidol 30 mg daily for 3 weeks, along with valproate preparation 1500 mg daily. He was started on clozapine as he was nonresponsive to Lithium in previous episodes and did not consent to starting Electroconvulsive Therapy (ECT). He responded adequately to 100 mg clozapine and 1500 mg valproate preparation and remission happened within 2 weeks of starting clozapine. Clozapine can be a good option for resistant mania and further RCT based evidences will strengthen the options in treating resistant mania.

## 1. Introduction

Bipolar mood disorder is a mental disorder with a lifetime prevalence rate of about 1% in the general population [1]. Recent advances in research have provided numerous effective treatments for bipolar mood disorder. However, there are still a proportion of individuals who suffer from bipolar mood disorder that is resistant to standard treatment. The concept of treatment resistance in bipolar disorder is clinically familiar but lacks a standard definition [2]. The term is not clarified with regard to nonresponsiveness to one or more standard treatments, at what dosages and what phases of bipolar disorder. Treatment resistance in bipolar disorder should always be based on a specific phase of treatment: mania or depression and acute or maintenance [3]. Reporting clozapine responsive mania that was not responding to two previous consecutive atypical antipsychotics and one typical antipsychotic along with valproate that can create benchmark data in a country like Bangladesh was aimed at.

## 2. Case Report

Mr. OG, 17-year-old young man, unmarried, student, currently unemployed, with average intelligent level, hailing from the rural background with lower economic background, was admitted into psychiatry inpatient department of Bangabandhu Sheikh Mujib Medical University with the symptoms of mania for the last 15 days. He was hospitalized three times previously with the same complaints over the last 2 and half years. He first presented with the complaints of irritability, inflated self-esteem, talkativeness, decreased need for sleep, increased goal directed activity, delusion of grandiosity in the form of grandiose identity, and excessive spending, 2 and half years earlier. He was diagnosed as a case of bipolar mania and was functioning with medications. Unfortunately he experienced frequent relapse due to different factors; among them one was noncompliance. In this recent episode he fulfilled the diagnostic criteria of mania and started receiving sodium valproate 1500 mg daily and Risperidone 4 mg daily as he was responding to those medications previously and was maintaining his daily active life. Risperidone was gradually uptitrated to 12 mg daily along with the valproate dose and followed up to 4 weeks. But he did not respond to the medications in any form covering the symptom intensity or number of symptoms and then Olanzapine was started and uptitrated to 30 mg daily along with stopping Risperidone with downtitration with the same valproate dose. He was followed up for the following 3 weeks but still he was not responding to the medications. Then he started Haloperidol uptitrated to 30 mg daily with coverage of procyclidine along with cross tapering of the Olanzapine and similar dose of valproate. Again he was followed up for the following 3 weeks for improvements but unfortunately he did not respond. As patient was not responding to Lithium in previous episodes with adequate blood level and adequate duration, we considered Lithium with least possibility to choose for Mr. OG and Lithium was not started. We tried to convince the legal guardians for availing Electroconvulsive Therapy (ECT) but unfortunately failed. So, we considered clozapine with cross tapering of Haloperidol. Clozapine was started with 25 mg at night and rapid titration was considered compared with the usual protocol as he was under close supervision and previously nonresponsive to high dose antipsychotics. Clozapine was titrated up to 100 mg daily in divided dose within one week and he was observed closely. Fortunately, he did not have any noticeable side effects in clozapine therapy in both initiation and continuation. Now, authors see the hope of light and Mr. OG responded significantly to clozapine 100 mg daily in divided doses and remission of symptoms happened within the following week. Treatment continued with clozapine 100 mg daily in divided doses and 1500 mg valproate preparations. He was discharged from hospital for further observation with full psychoeducation and active daily life program. Ethical issues were maintained accordingly and written informed consent is taken for publication of the report from the patient.

## 3. Discussion

Though a common occurrence, identification of treatment resistant bipolar disorder cases is challenging as there seems to be a lack of consensus about its definition [2]. The general agreement would be that a lack of response or rather no response to standard treatment therapies that are known to be effective would best define treatment resistant bipolar disorder [2, 3]. Electroconvulsive Therapy (ECT) and clozapine are nonstandard treatment options that have been suggested for use in treatment resistant mania [3]. Clozapine is the first atypical antipsychotic agent that acts on various neurotransmitters, and it appears to be an effective treatment. However, judicious use of clozapine is required due to the lack of thorough studies and the serious side effects associated with its use. In the case of Mr. OG, he failed to respond to two second-generation (Risperidone and Olanzapine) antipsychotics and one first-generation (Haloperidol) antipsychotic with their maximum doses along with mood stabilizer (Valproate) for the treatment of mania. There have been a few guidelines that have suggested using clozapine in cases of bipolar mania. According to the Canadian Network for Mood and Anxiety Treatments (CANMAT) guidelines for bipolar disorder, treatment with clozapine for acute bipolar mania is a third-line option and should be reserved for use in treatment resistant patients due to safety concerns and the lack of double blinded randomized controlled trials [4]. The American Psychiatric Association's guidelines state that clozapine may be particularly effective in refractory cases of bipolar mania [5]. Although large-scale studies have been few and far between, there are certain studies that reveal clozapine to be effective in cases of treatment resistant mania. In a study by Green et al., it was found that 17 out of 22 treatment-refractory bipolar disorder manic types with psychotic features showed at least 20% improvement in the Brief Psychiatric Rating Scale, Young Mania Rating Scale, and the Clinical Global Impressions Scale after treatment with clozapine [6]. Similarly, there have been other studies that show clozapine's effectiveness in treating refractory affective illness [7, 8]. Correspondingly, Mr. OG responded well after starting with clozapine together with mood stabilizer valproate preparation. Clozapine was gradually titrated to a total dose of 100 milligrams per day and within one week of starting as he was under close observation in the hospital ward and nonresponsive to other several antipsychotics. He did not experience any noticeable side effects of clozapine in initiation as well as continuation of this treatment. Mr. OG responded significantly and was discharged from hospital after further one-week follow-up. A recent study has found that rapid titration of clozapine is safe and is associated with a shorter hospital stay as compared to slow titration in the treatment of refractory bipolar disorder [9] and in the reported case rapid titration method was done with a cautious approach. To date, the management of treatment resistant bipolar disorder remains a challenge. As mentioned earlier, the effectiveness of clozapine in treating Mr. OG was consistent with the findings of current literature. Thus, it is worthwhile to consider the use of clozapine as a treatment modality in resistant cases. It is with much hope that further studies will be done in this area so that better understanding can be obtained, and this will assist clinicians in better managing patients with treatment resistant mania. Moreover, case report of Mr. OG will encourage the physicians to consider clozapine in resistant mania in a country like Bangladesh and will create benchmark data regarding clozapine in treatment resistant mania.

## 4. Conclusion

There is still paucity of standardized guideline for using clozapine in resistant mania. From the reported case it can be inferred that clozapine can be good option for resistant mania and the case may contribute to standardizing a guideline for resistant mania with further evidence from further strong methodological studies.
